# Can Intrapartum Ultrasonography Improve the Placement of the Vacuum Cup in Operative Vaginal Deliveries?

**DOI:** 10.3390/tomography9010019

**Published:** 2023-01-27

**Authors:** Rocio Garcia-Jimenez, Irene Valero, Carlota Borrero, Jose Antonio Garcia-Mejido, Ana Fernandez-Palacin, Rosa Serrano, Jose Antonio Sainz-Bueno

**Affiliations:** 1 Obstetrics and Gynecology Department, Juan Ramon Jiménez Hospital, 21005 Huelva, Spain; 2 Obstetrics and Gynecology Department, Valme University Hospital, 41014 Seville, Spain; 3 Obstetrics and Gynecology Department, Faculty of Medicine, University of Seville, 41009 Seville, Spain; 4 Biostatistics Unit, Department of Preventive Medicine and Public Health, University of Seville, 41009 Seville, Spain; 5 Obstetrics and Gynecology Department, Jerez University Hospital, 11407 Jerez de la Frontera, Spain

**Keywords:** operative vaginal delivery, vacuum-assisted delivery, intrapartum ultrasonography, transabdominal intrapartum ultrasound, transperineal ultrasound, fetal head position

## Abstract

Although the fetal head position has traditionally been evaluated by digital examination (DE), it has a failure rate ranging between 20 and 70%; hence, intrapartum transabdominal ultrasonography (TUS) has become relevant. We aimed to evaluate the utility of the TUS to identify the fetal head positions in vacuum-assisted deliveries. We performed a prospective observational study including 101 pregnant patients in active labor who required a vacuum-assisted delivery. The fetal head position was assessed by a DE and a TUS prior to vacuum cup placement. After delivery, the optimal vacuum cup placement was evaluated as the distance between the chignon and the flexion point ≤2 cm. The general concordance rate between the DE and TUS was 72.2%, with the poorest concordance rate for occiput posterior positions at 46.1%. In five cases (4.9%), it was not possible to determine the fetal head position through the DE. The correlation was higher in low and medium planes, with 77% and 68.1% concordance rates, respectively, while it was lower in high planes (60%). In 90.1% of cases, the vacuum cup placement was optimal. Our findings show that intrapartum transabdominal ultrasonography is a useful technique to identify the fetal head position allowing optimal placement of the vacuum cup necessary for correct vacuum-assisted delivery.

## 1. Introduction

Operative vaginal birth is a procedure which entails some risks, but it is associated with a decrease in neonatal and maternal morbidity and mortality rates when performed correctly [1,2,3]. Hence, it is important to determine the precise position of the fetal head to properly place a vacuum or forceps, since the incorrect placement of these obstetrical instruments increases the risk of failure and may lead to fetal injuries [4,5].

Traditionally, the fetal head position has been evaluated by digital examination (DE). However, it has been established that DE is quite limited in the evaluation of the position and station of the fetal head, with a failure rate ranging between 20 and 70% when compared with an ultrasound as the gold standard [6]. This is more frequent for anomalous positions, such as the occiput transverse or posterior, especially in the presence of caput succedaneum or asynclitism, which are situations that are usually associated with stalled labor and require medical intervention more often [6,7,8,9]. Thus, intrapartum ultrasonography has become relevant. Several authors have shown that ultrasonography, both by itself and in combination with DE, is superior to DE alone in determining the fetal head position [6,7,8,9,10,11,12,13,14]. A randomized study revealed that ultrasonography evaluation combined with DE before an assisted vaginal delivery was significantly more accurate in the diagnosis of the fetal position than DE alone, with a failure rate of 1.6% vs. 20.2%, respectively [15].

Our main objective was to evaluate the utility of transabdominal ultrasonography (TUS) to identify the fetal head position and enable the correct cup placement in vacuum-assisted deliveries.

## 2. Materials and Methods

A prospective observational study was carried out between December 2021 and April 2022, including 101 at-term pregnant patients, who were admitted to the Labor and Delivery Ward at Valme University Hospital, were in active labor, and required an operative vaginal birth. The study received the approval of the local bioethics committee.

The patients were consecutively recruited at the time of the assessment for a vacuum-assisted delivery if they met the inclusion criteria, which were primiparous women with a singleton at-term pregnancy (37–42 weeks) in the second stage of labor, with ruptured membranes, a longitudinal fetal situation, and cephalic fetal presentation. The exclusion criteria were severe maternal or fetal diseases and if the fetal delivery was achieved by caesarean section, forceps delivery, or spontaneous vaginal delivery even when there was a previous intention for vacuum-assisted delivery.

**Figure 1:**
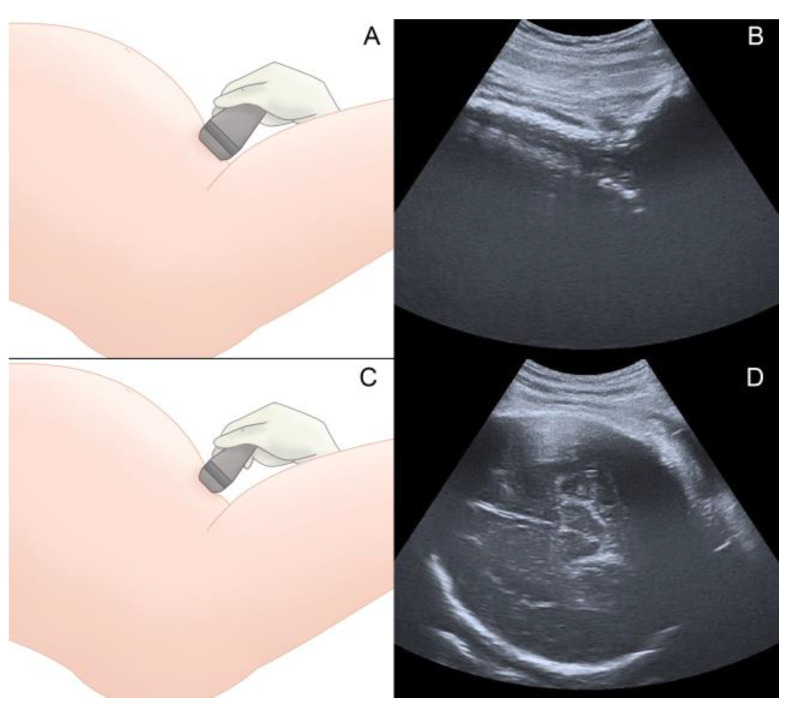
(**A**) Transabdominal ultrasonography with the probe placed longitudinally and tangentially to the abdomen. (**B**) Identification of the fetal cervical spine and occipital bone in a sagittal transabdominal plane. (**C**) Transabdominal ultrasonography with the probe placed transversely at the suprapubic region. (**D**) Visualization of the brain structures in a transverse transabdominal plane.

**Figure 2:**
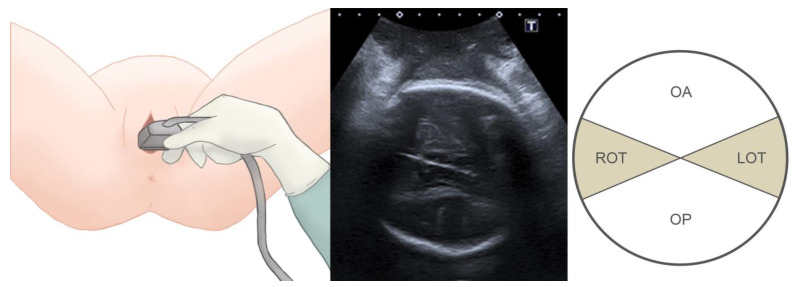
(**Left**) Transperineal ultrasonography to evaluate the fetal head position. (**Center**) Visualization of the midline angle plane to assess the fetal head position. (**Right**) Classification of the fetal occiput positions (OA: occiput anterior; LOT: left occiput transverse; OP: occiput posterior; ROT: right occiput transverse).

**Figure 3:**
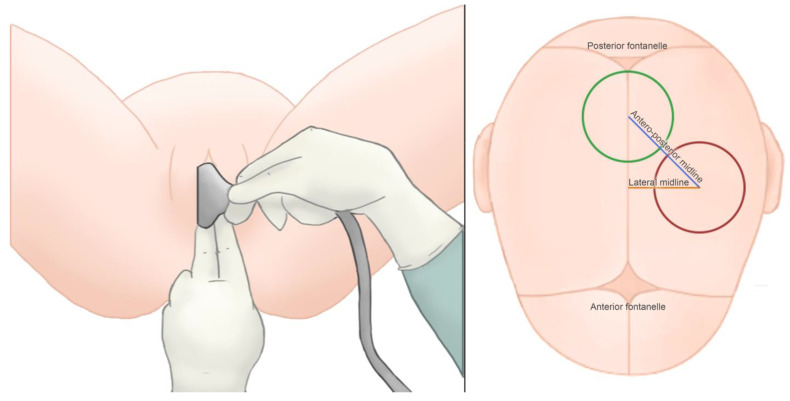
(**Left**) Ultrasound assistance for vacuum cup placement, with probe placed in the sagittal transperineal plane. (**Right**) Measurement of the distance between the chignon (red) and flexion point (green).

The patients were first assessed when a vacuum-assisted delivery was indicated. Prior to the application of the vacuum cup, DE was performed to assess the fetal head position according the ACOG guidelines [16]. Next, a suprapubic transabdominal ultrasonographic (TUS) evaluation was carried out using a Toshiba Famio 8 (Tokyo, Japan) unit with a convex 3.75 MHz probe, following the ISUOG guidelines for intrapartum ultrasound [17]. The probe was first placed longitudinally and tangentially at the suprapubic region of the maternal abdomen to identify the fetal cervical spine and occipital bone and then transversely to assess the fetal head position, using the fetal orbital region, cervical spine, cerebral midline, and cerebellum as references [10,18,19], as shown in Figure 1. Next, transperineal ultrasonography was performed transversely, in the midline angle plane to identify the brain structures to complete the assessment of fetal head position (Figure 2). Lastly, the probe was placed in the sagittal transperineal plane (Figure 3) to measure the angle of progression (AOP) to assess the level of the descent of the fetal head in the maternal pelvis [17,20].

Vacuum-assisted delivery was performed using a Malmström cup. The vacuum cup was considered to be optimally placed when the center of the chignon was 6 cm posterior from the anterior fontanel on the sagittal suture. After the delivery, a midwife used a transparent plastic sheet to mark the distance between the center of the chignon and the flexion point, measuring two deviations, in centimeters (cm), the anterior–posterior and the lateral midlines (Figure 3). The vacuum cup placement was considered to be optimal when the distance between the center of the chignon and the flexion point was 2 cm or less.

Statistical analysis was carried out using the statistical package IBM SPSS statistics 22 (IBM, Armonk, NY, USA). The quantitative variables were described as the means and standard deviations, while the qualitative variables were described as the frequencies and percentages. The normality of the data was contrasted using the Shapiro–Wilk test. Comparisons between the study groups were performed using Student’s t-test for independent samples if the data were normally distributed, and the Mann–Whitney U-test was performed for nonnormally distributed data. The significance level was set at *p* < 0.05.

## 3. Results

A total of 106 patients were included in the study. There was a loss of five patients due to incomplete data; thus, the number of patients composing the final sample was 101. The mean maternal age was 31.5 ± 5.92 years, while the mean gestational age was 39.57 ± 1.55 weeks. The most frequent indication for vacuum-assisted birth was a prolonged second stage of labor (68.3%). A total of 11.8% of the patients had a previous cesarean section, and the induction rate was 26.7%. The rest of the sociodemographic and obstetric parameters are displayed in Table 1.

In Table 2, we can see the neonatal outcomes. The mean Apgar scores at 1 and 5 min were 8.8 ± 1.051 and 9.96 ± 0.268 respectively, with a mean pH umbilical cord of 7.244 ± 0.782. Admission to the NICU was required in two cases (1.9%), and there were three cases of perinatal morbidity: two cases of head laceration (1.9%) and one case of head trauma (0.9%).

Table 3 displays the results of the accuracy of the DE. In the first column, we can see the prevalence of the various position as detected by the TUS in juxtaposition with those detected by the DE in column 2. In column 3 and 4, we show how many of these positions identified by the DE were correct and incorrect, respectively. Out of the 96 positions identified by the DE, 74 of them (77.1%) were correctly identified, according to TUS, whilst 22 of them (22.9%) were incorrect. Assuming the ultrasound as the gold standard, the detection rate (DR) of the DE was the highest in the occiput anterior positions (100%), although the DE overestimated its presence by almost twofold (DE 17.8% vs. US 9.9%). The next highest accuracy of the DE was for the left occiput anterior (84.6%) and right occiput transverse (81.0%) positions, while the poorest agreement between the DE and the TU was shown for the occiput posterior positions (46.2%). In five cases (4.9%), it was not possible to determine the fetal head position through the DE. The general DR of the DE was 73.3%. The total false negative rate (FNR) of the DE was 26.7%, with the highest percentage for the occiput posterior positions with a 53.9% FNR.

The agreement between the DE and the TUS depending on the level of descent of the fetal head in the maternal pelvis is shown in Table 4. The accuracy was higher in the low and medium planes, with 77% and 68.1% correlation rates, respectively, while it was lower in the high planes (60%).

Regarding the vacuum cup placement, there was a lateral deviation from the flexion point of 0.8 ± 0.5 cm. The mean distance between the center of the chignon and the flexion point was 1.6 ± 1.0 cm. In 90.1% (91/101) of the cases, the distance to the flexion point was less than 2 cm. The occiput posterior positions comprised six out of the ten cases in which the distance was more than 2 cm. Although in 9.9% (10/101) of the cases, there was a suboptimal placement due to a distance between the chignon and the flexion point higher than 2 cm, none of these was placed anterior to the flexion point.

## 4. Discussion

Operative vaginal birth is a procedure associated a higher risk of fetal injury and perinatal mortality when incorrectly performed [4,5], which may end up in a failed assisted delivery, which can prolong the indication–birth time interval and increase the risk of maternal and fetal injuries [21,22,23,24,25,26,27]. In particular, vacuum-assisted deliveries have an estimated failure rate of between 4 and 23%, which is associated with an increased risk of maternal and fetal morbidity and mortality, such as a 3.5-fold risk of postpartum hemorrhage [28,29]. Among the causes of failure of vacuum deliveries, the most frequent is the incorrect application of the vacuum cup away from the flexion point, which has been reported in up to 50% of cases [2,30,31]. Mola et al. [3] established that in failed vacuum-assisted deliveries there is a 4.5-fold chance of cup placement in a deflexed position, which can result in low APGAR scores, severe scalp, and admission to NICU. These results are similar to those published by Chadwick et al. [32], which showed that an incorrect vacuum application was associated with an increase in subgaleal hematomas, as well as those by Teng et al. [33], who found that paramedian cup placements was a risk factor for neonatal scalp, along with the time to place the cup and the second phase duration.

Thus, it is crucial to determine the exact fetal head position to safely assist an operative vaginal birth, as a properly performed operative vaginal delivery has been associated a decrease in the morbidity and mortality for both the mother and newborn [1,2,3]. Although the fetal position has traditionally been evaluated through the DE, several studies have revealed its accuracy to be quite limited [6,7,8,9,10,11,12,13,14,15]. It is considered to be a subjective form of assessment, and its error rate has been found to be up to 30% [11], 46% [8], and 61% in the first stage of labor and 31% in the second [12]. Akmal et al. found similar results, with an error rate ranging between 26.6 and 34% [9]. Dupuis et al. [34] conducted a study in which they used a labor simulator, finding that the DE had a global error rate of 36–80%, failing to correctly identify the descent of the fetal head in medium and high stations at 34% and 67%, respectively.

In this light, the appearance of intrapartum ultrasonography seems promising, as its utility has been widely proven. It is useful to predict whether a delivery will occur spontaneously or with obstetric assistance, and it can even predict the success of said intervention [35,36,37,38,39,40]. Concerning vacuum-assisted deliveries, a randomized study performed by Wong et al. [41] showed that, although no differences were found between ultrasonography and DE alone regarding the assessment of fetal head position, the use of ultrasonography is helpful to ensure the placement of the vacuum cup closer to the flexion point. A study conducted by Haiki et al. [42] found that vacuum placement was not associated with obstetrician expertise in finding cranial sutures, and in 28.5% of cases, ultrasonography detected an incorrect cup placement, resulting in modification of the placement, which was then successful in 92.5% of cases. Our results were similar to this, as we found a global error rate of 27.8% for the DE, with a higher discrepancy for occiput posterior positions and medium and high stations. It is worth noting that despite the imprecision of the DE, the use of ultrasonography as the gold standard in our study helped to correctly identify the fetal position in all cases, achieving a correct vacuum cup placement in 90.1% of cases in which the distance between the flexion point and the chignon was less than 2 cm.

Nonetheless, our study also had its limitations, with the small sample size as the main issue. Future studies should follow this line of research with a larger number of participants recruited for a longer period of time. It would also be of interest to include a control group with patients assessed only by digital examination, comparing the outcomes with a group assessed by ultrasound, thus measuring how the use of ultrasonography might improve the outcome in vacuum deliveries.

Nevertheless, our findings show that intrapartum transabdominal ultrasonography is a useful technique to identify the fetal head position allowing the optimal placement of the vacuum cup necessary for correct vacuum-assisted delivery.

## Figures and Tables

**Table 1:** Sociodemographic and obstetric parameters.

Parameter	Mean ± SD or *n* (%)
Mean maternal age	31.50 ± 5.92
History of cesarean section	12 (11.8%)
Gestational disease	18 (17.8%)
Gestational diabetes	3 2.9%)
Hypertensive state of pregnancy	3 (2.9%)
Intrauterine growth restriction	7 (6.9%)
Others	7 (6.9%)
Gestational weeks at delivery	39.57 ± 1.55
Induced deliveries	27 (26.7%)
Chronological prolonged pregnancy	6 (5.9%)
Ruptured membranes	8 (7.9%)
Intrauterine growth restriction	6 (5.9%)
Hypertensive state of pregnancy	3 (2.9%)
Others	4 (3.9%)
Epidural analgesia	101 (100%)
Number of operative deliveries (vacuum)	101 (100%)
Indication of operative delivery	
Prolonged second stage	69 (68.3%)
Others	32 (31.7%)
Tear of cesarean section scar	3 (2.9%)

The results are displayed as means and standard deviations (SD) or frequencies and percentages.

**Table 2:** Neonatal outcomes.

Parameter	Mean ± SD or *n* (%)
Newborn sex (females)	47 (46.5%)
Newborn weight in grams	3.332 ± 422.44
APGAR at 1 min	8.80 ± 1.051
APGAR at 5 min	9.96 ± 0.268
Newborn umbilical artery pH	7.24 ± 0.782
Perinatal mortality	0 (0%)
Perinatal morbidity	3 (2.9%)
Head laceration	2 (1.9)
Head trauma	1 (0.9%)
Admission to NICU	2 (1.9%)

The results are displayed as means and standard deviations (SD) or frequencies and percentages.

**Table 3:** Accuracy of the digital examination for assessing the fetal head position in comparison to transabdominal ultrasonography.

Fetal Head Position	Identified by TUS	Identified by DE	Correct Position (DE)	Incorrect Position (DE)	DE DR (DE/TUS)	DE FNR
Direct occiput anterior	10 (9.9%)	18 (17.8%)	10/18 (55.6%)	8/18 (44.4%)	100.0% (10/10)	0.0% (0/10)
Right occiput anterior	19 (18.8%)	11 (10.8%)	11/11 (100%)	0/11 (0.0%)	57.9% (11/19)	42.1% (8/19)
Right occiput transverse	21 (20.7%)	23 (22.7%)	17/23 (73.9%)	6/23 (26.1%)	81.0% (17/21)	19.1% (4/19)
Left occiput anterior	26 (25.7%)	25 (24.7%)	22/25 (88.0%)	3/25 (12.0%)	84.6% (22/26)	15.4% (4/26)
Left occiput transverse	12 (11.8%)	11 (10.8%)	8/11 (72.7%)	3/11 (27.3%)	66.7% (8/12)	33.3% (4/12)
Direct occiput posterior	13 (12.8%)	8 (7.9%)	6/8 (75.0%)	2/8 (25.0%)	46.2% (6/13)	53.9% (7/12)
Not possible	0 (0%)	5 (4.9%)				
Total			74/96 (77.1%)	22/96 (22.9%)	73.3% (74/101)	26.7% (27/101)

DE: digital examination; TUS: transabdominal ultrasound; DR: detection rate; FNR: false negative rate.

**Table 4:** Accuracy of the digital examination in comparison to the transabdominal ultrasonography depending on the fetal head’s level of descent.

Level of Descent of the Fetal Head (Hodge’s Planes; Lee’s Stations; Angle of Progression)	DE	DE DR (DE/TUS)
High (I/II; −1/−3; AOP < 116°)	5 (4.9%)	3/5 (60%)
Medium (III; 0; AOP = 116°)	22 (21.7%)	15/22 (68.1%)
Low (IV ;+3; AOP > 148°)	74 (73.2%)	56/74 (75.7%)
Total	101 (100%)	74/101 (73.3%)

## Data Availability

Not applicable.
